# Bismuth Soup and X-Rays in Œsophageal Obstruction

**Published:** 1908-11-14

**Authors:** 


### BISMUTH SOUP AND X-RAYS IN ŒSOPHAGEAL OBSTRUCTION.

The diagnosis of œsophageal obstruction is not difficult when it has reached an advanced degree, but it is by no means easy in earlier stages. The nature of the obstruction is not absolutely easy of determination at any stage. The commonest causes by far are, first, squamous-celled carcinoma, starting primarily in the œsophagus; secondly, aneurysm of the descending thoracic aorta obstructing the œsophagus from without; and thirdly, columnar-celled or spheroidal-celled carcinoma, starting at the cardiac end of the stomach and thence extending upwards and involving the lower end of the œsophagus.

The fact and the site of œsophageal obstruction are both determined ultimately in most cases by the passage of an œsophageal bougie. There would be no great objection to the bougie in all cases were it not for the possibility of the obstruction being aneurysmal; but so difficult is it to exclude aneurysm before a bougie is passed that again and again the operation has to be done tentatively without complete freedom from a fear that a sudden gush of blood from a ruptured aneurysm may result.

This being so, it is clear that other means of determining the site and nature of œsophageal obstructions are to be welcomed, and such means are to be found in the *x*-rays after the patient has partaken of bismuth soup, or whilst he is partaking of it. The bismuth may be given in relatively huge quantities with impunity if the carbonate is employed. The nitrate or subnitrate is less safe, apparently owing to the presence of nitrites in the salt. The carbonate or oxycarbonate is free from danger, and it produces no constipation, notwithstanding text-book remarks to the contrary.

The most convenient method of administering it is by mixture with bread and milk; one and a half ounces of the carbonate are added to each half-pint of the bread and milk, and mixed up till the whole is of the consistence of thick porridge. Instead of giving it in bread and milk the bismuth may be suspended in ordinary thick mucilage, but the most convenient form is the former, which the patient eats in the ordinary way with a spoon.

Each mouthful of bismuth food can be watched in its passage down the œsophagus. The time it should take in passing, and the manner in which it goes down in a normal person, have been fully studied and recorded; any departure from the normal can be detected at once. In the case of œsophageal obstruction the bolus of thick bismuth food will be seen to travel down to a certain point and then suddenly stop. If the obstruction is not extreme a small amount of the bismuth will be seen gradually percolating through it, finally detaching itself from the main mass and passing on to the stomach. In some cases it is even possible to watch the rhythmical contractions of the œsophagus as it tries to drive the bismuth food through the obstruction, in which case there will presently be a reversal of the peristaltic wave, and the food will be seen regurgitating far up into the neck again.

Not only does the method afford an ocular demonstration of the fact that there is obstruction, and of its precise site—it also assists greatly in distinguishing obstruction by growth from obstruction by aortic aneurysm. An aneurysm which can obstruct the œsophagus will seldom fail to cast an *x*-ray shadow of itself, which a carcinoma of the œsophagus would not.

It is undoubtedly true that such an aneurysmal shadow might be lost in the cardiac shadow if the patient were screened only in the antero-posterior axis; but nowadays, especially for œsophageal work, there are arrangements for examining the patient in the vertical position upon a stool which can be rotated to allow of examination in each and every axis; the bismuth-food movements are followed most easily when the patient is sitting sideways to the *x*-rays, and in this position an aneurysm of the descending thoracic aorta is less likely to escape detection than it is in any other.

